# Case Report: Treatment policy for female adolescents in the grip of chronic suicidality

**DOI:** 10.3389/frcha.2024.1384439

**Published:** 2024-05-14

**Authors:** M. Van de Koppel, S. Y. M. Mérelle, Y. A. J. Stikkelbroek, P. T. Van der Heijden, J. Spijker, A. Popma, D. H. M. Creemers

**Affiliations:** ^1^ Depression Expertise Center for Youth (DEC-J) at GGZ Oost Brabant, Mental Health Care Institution, Oss, Netherlands; ^2^ National Dutch Suicide Prevention Center, 113 Suicide Prevention, Amsterdam, Netherlands; ^3^ Clinical Child, Family and Education Studies, Social and Behavioral Sciences, Utrecht University, Utrecht, Netherlands; ^4^ Reinier van Arkel Group, Mental Health Care Institution, ‘s-Hertogenbosch, Netherlands; ^5^ Pro Persona, Mental Health Care Institution, Nijmegen, Netherlands; ^6^ Experimental Psychopathology and Treatment, Behavioral Science Institute, Raboud University, Nijmegen, Netherlands; ^7^ Academic Medical Centre, Amsterdam UMC, Amsterdam, Netherlands; ^8^ Child and Youth Department, Dutch Association for Psychiatry, Amsterdam, Netherlands

**Keywords:** chronic suicidality, adolescence, youth mental health care, suicidal behavior, case description, guidelines

## Abstract

**Introduction:**

Within the Dutch clinical field of specialized mental health care for youth, an increasing subgroup of female adolescents with severe chronic suicidal behavior is recognized. This group was also identified in a Dutch psychological autopsy study among 35 relatives of adolescents (aged 10–19 years old) who died by suicide. There seems to be a lack of knowledge and consensus how to treat this severe chronic suicidal behavior, resulting in stagnation of care and growing demoralization among patients, parents, and mental health care providers. The aim of this paper is to describe characteristics of the suicidal process, to identify challenges experienced in providing mental health care for this subgroup, and to formulate preliminary recommendations.

**Method:**

A case description from the psychological autopsy study and a review of the relevant literature.

**Results:**

The persistent suicidal threat and the resulting despair of the patient and their parents are forcing the mental health care provider into an impasse: the primary focus of treatment slowly moves to guarantee the patient's safety, which leaves the treatment of underlying problems underexposed. Due to the chronicity of the suicidal ideation and behavior in a phase in which identity formation and developing cognitive and emotional regulation skills are important developmental tasks, we identify a risk of developing a suicidal identity.

**Discussion:**

Based on expert knowledge, we make recommendations on (1) treating suicidality as a transdiagnostic phenomenon with its own meaning and function, (2) implementing treatment considerations promoting the autonomy, (3) aiming at continuity of care and prevention of repeated patient referrals by creating a multidisciplinary network of care providers, and (4) making chronic suicidality tolerable for the care provider.

**Conclusion:**

We propose preliminary practical recommendations in our quest for optimal mental health care for chronically suicidal adolescents.

## 1 Introduction

Within the Dutch clinical field, an increasing subgroup of female adolescents with severe chronic suicidal behavior is recognized by care providers of youth mental health care institutions and secure residential youth care (1, 2). Suicide is one of the leading causes of death among adolescents worldwide. The most significant rise in the number of suicides during the lifespan occurs during the phase from early adolescence to young adulthood (3). In the Netherlands, however, there was a sharp increase in the number of suicides among teenagers in 2017 compared to previous years (4). The Dutch Ministry of Health, Welfare and Sport therefore requested the Dutch Suicide Prevention Center (113 Suicide Prevention) to conduct an in-depth investigation into the backgrounds of these suicides. The relatives of 35 adolescents (aged 10–19 years old) deceased by suicide were included in a psychological autopsy study to gain more insight into the circumstances under which these adolescents died by suicide (5, 6). Interviews and questionnaires were administered to the parent(s), a peer, a healthcare provider, and teachers. In line with the observations of the Dutch clinical field, within the psychological autopsy study a subgroup of female adolescents with severe chronic suicidal behavior was identified. This group consisted of seven intelligent girls who struggled with insecurity, perfectionism, fear of failure, and performance pressure. Following a childhood without severe (psychological) problems, they exhibited a downward spiral in development from high school onwards, marked by depressive episodes, anxiety, emotional regulation problems, eating disorders, and severe, chronic suicidal behavior. Often, there was a history of repeated admissions to crisis units, with their social networks increasingly consisting of young people with psychological problems. Significantly, both parents and therapists experienced a sense of powerlessness, as the psychological state of the patient worsened despite all efforts (5). There seems to be much ambiguity concerning the treatment and approach for chronic suicidal behavior of this subgroup, possibly resulting in stagnation of care and increasing demoralization among patients, parents, and mental healthcare providers. A case from the 2017 Dutch psychological autopsy study is described for the purposes of illustration.

Note: None of the authors had any treatment relationship with the patient described.

### 1.1 Case description

Patient A was an 18-year-old girl who died by suicide. At the time of her death, the DSM-5 primary classification was an avoidant personality disorder with comorbid persistent depressive disorder, anorexia nervosa, and an unspecified anxiety disorder. She had received mental healthcare from various institutions since the age of 14. Initially, she started with supportive appointments with a social worker and cognitive behavioral therapy in outpatient mental health services. This was followed by nine months of clinical treatment in an inpatient mental healthcare facility and eleven months of outpatient treatment aimed at reducing anxiety and depressive symptoms, suicidality, and eating disorders. During this period, there were repeated admissions to the crisis unit as a result of severe suicide attempts. There was an inadequate response to treatment, and the care provided became increasingly characterized by the safety management of the suicidality by applying interventions aimed at repression. For a year, efforts were made to register the patient for (outpatient) clinical treatment programs, but she was repeatedly rejected due to the complexity of her problems. The repeated admissions to the crisis unit usually resulted in a temporary reduction of suicidal behavior only. As soon as the patient was expected to take on more responsibility associated with the discharge of the crisis admission (for example, the freedom to go wherever she wanted to go, the responsibility to communicate distress without the twenty-four-hour availability of a team of professionals), the parents and mental health care providers observed a marked increase in suicidal behavior.

#### 1.1.1 Information from the parents

The patient grew up in a stable family as the middle child of three daughters. There were no adverse childhood experiences. She had friends, was never bullied, and had good academic achievements. However, there was a predisposition to insecurity and perfectionism. She wanted to meet everyone's expectations and to fit in all peer groups, but it was difficult for her to bear this (self-imposed) pressure. She would withdraw when she was sad, and exhibited outbursts of rage when she was angry.

From the age of 14, she began to develop psychological problems. There was evidence of anxiety due to performance pressure, (social) insecurity, an increasing need for control, and self-harm. She also developed restrictive eating patterns and became increasingly depressed and suicidal. She stopped attending school in her junior year. According to the parents, the increasing responsibility that comes with (young) adulthood made her anxious. The parents reported that they struggled with openly discussing the suicidal ideations, fearing it would trigger their daughter's suicidal behavior. Additionally, the parents stated that they frequently felt insufficiently seen and heard in their perspectives, needs and feelings of powerlessness during the entire treatment process of their daughter.

#### 1.1.2 Information from the former therapist (clinical psychologist)

The nine-month clinical treatment consisted of creating a crisis plan, pharmacological treatment, cognitive behavioral therapy, eye movement desensitization and reprocessing (EMDR), psychomotor therapy, and therapeutic guidance for the parents with the main goals to reduce suicidal behavior and suicidal ideation and to reduce anxiety and depressive symptoms. The therapist described that due to the enduring nature of the suicidality, ensuring her safety became the primary focus of treatment. During the treatment process, the professionals and the parents developed different perspectives. The parents advocated for more safety measures and an intensification of assistance and care. By contrast, the professionals argued for fostering autonomy. Despite the efforts of the treatment team, the patient's suicidal behavior continued to increase, and ultimately 24-hour supervision was necessary to ensure her safety.

#### 1.1.3 Information from a peer (friend of patient A)

The patient was sensitive, reserved, and fearful of the responsibilities that accompany growing up. She followed posts about eating disorders and suicidality on social media, in the process creating a new identity online. The peer stated that being mentally ill became “who she was”, and her contact with fellow patients and social media helped to maintain that identity.

## 2 Reflection

The case description illustrates the persistent suicidal threat and the feelings of hopelessness of the patient and her parents, which may have forced the healthcare providers into an impasse: the primary focus of treatment became safety management and the treatment of underlying problems shifted to the background. Although clear guidelines about the assessment and treatment of adolescents with suicidal behavior exist (7), there is a need to increase the evidence base for chronic suicidality. The Dutch Multidisciplinary Guideline for Diagnostics and Treatment of Suicidal Behavior (8) offers valuable recommendations for the mental healthcare of adults:

(1) Chronic suicidal behavior needs to be taken seriously at all time, but be cautious in reacting to this behavior with immediate safety measures.
(2) Look for the source and reasons for the continuation of the psychological distress in a long-term engagement with the patient.
(3) Avoid becoming a victim of the patient's suicidal threats; continue to strive for change and address and appeal to the patient's autonomous capacity.
(4) Be aware of responding out of countertransference, especially feelings of powerlessness and aggression;
(5) Make regular use of intervision or supervision.
(6) Remain vigilant during acute phases of chronic suicidal behavior and consider intensifying care in the case of *acute risk* in addition to the *chronic risk*.
(7) Ensure support at every level of the organization when there is a severe threat of suicide and homicide.

However, these guideline seems to fall short in the adolescent phase of life, where autonomous capacity is still developing. There is often a family and social network around the patient that exerts significant pressure on mental healthcare providers to eliminate all suicide risks (9). Moreover, the dynamics of treatment between patient, parents and therapist are often complex due to mutual distrust arisen from a lack of shared and endorsed vision regarding the aim of the treatment. This may result in a risk that each person involved (patient, parents, or therapists) feels solely responsible for the patient's survival (9–11). As a result, the focus of the treatment is often on the management of suicidality and potentially lacks a sufficient basis for development. Before we proceed with additional recommendations, we will first clarify the meaning of chronic suicidality.

### 2.1 Chronic suicidality

Chronic suicidality can be described as the persistent or recurrent presence of suicidal thoughts, the formation of suicide plans, and/or undertaking suicide attempts (12, 13). Chronically suicidal adolescents seem to differ from acutely suicidal adolescents in several areas, since they show more severe emotion regulation problems (14), hopelessness (15), and tend to start self-harm at a younger age (16).

In the Dutch psychological autopsy study, Mérelle et al. (5) suggest the development of a *suicidal identity*. During adolescence, the formation of one's identity and the development of cognitive and emotional regulation skills are important developmental skills (17, 18). Hormonal changes occur simultaneously, along with emotional instability and increasing responsibilities. This may lead to a discrepancy between the required cognitive and emotional skills to cope with everyday challenges and the actual capabilities of the adolescent, which can result in feelings of being overwhelmed, depression, and anxiety.

Adolescents also tend to be more prone to all-or-nothing thinking and behavior (18), including suicidal behavior. Initially, suicidal tendencies are often egodystonic; in other words, they are not a part of one's *normal self* and lead to distress. However, as suicidality gradually becomes a functional coping mechanism by offering protection from the demands of an increasingly overwhelming world, for example, suicidal behavior becomes integrated into the normal self (egosyntonic) and will therefore evolve into a suicidal identity (19).

## 3 Recommendations

In addition to the existing Dutch guidelines, we make the following recommendations for the provision of mental health care for adolescents with chronic suicidality. In our experience, this subgroup is dominated by female adolescents, but the recommendations are applicable regardless of gender.

### 3.1 Treat suicidality as a transdiagnostic phenomenon

While suicidality is often seen as a characteristic of a disorder, it needs to be seen as a problem in its own right and requires adequate focus, evidence-based assessment [for example by using the Columbia Suicide Severity Rating Scale (20)] and evidence-based treatment. We have divided this recommendation into the following two steps.

Step 1: Look beyond suicide risk and safety

Too often, mental health care for suicidality is characterized by acute risk assessment and safety planning. This means that the focus of treatment is primarily on preventing a fatal outcome, rather than on recovery and growth (21). Intervention programs with sound scientific evidence for reducing suicidal ideation and/or behavior for adolescents are Dialectic Behavior Therapy for Adolescents (22), Cognitive Behavioral Therapy for Suicide Prevention (23), and Collaborative Assessment and Management of Suicidality (24, 25). Promising treatment interventions include the Attempted Suicide Short Intervention Program [ASSIP; (26)] and Youth-Nominated Support Teams [YST; (27)].

ASSIP is an additional form of psychotherapy that emphasizes the therapeutic alliance, psychoeducation, cognitive case conceptualization, and safety agreements. The patient receives outreach follow-ups from a treatment team for up to two years after the suicide attempt.

YST is an intervention focused on psychoeducation and social support, in which the adolescent selects an adult from their immediate environment as a support person after a crisis admission or suicide attempt. These support persons are trained and maintain weekly telephone contact with the YST team for three months.

However, an impasse is often experienced with the subgroup under consideration, in which no constructive therapeutic alliance can be formed and there is insufficient support to implement the abovementioned interventions. In such cases, Step 2 below might be indicated.

Step 2: Decelerate and tolerate

This step is about slowing down and tolerating the suicidal threat; it sometimes implies that therapists should refrain from active interventions, such as controlling and restrictive safety measures and crisis admissions, in order to avoid reinforcement of functional suicidal behavior and strengthening of the suicidal identity.

A therapeutic approach focused on providing reassurance and holding, such as mentalization-based treatment (MBT), and schema therapy, should be at the core of this process of decelerating. This is characterized by a safe and warm treatment relationship, which devotes attention to accepting the negative affect by understanding, identifying, and validating it without actively intervening. Ensuring safety through the use of controlling and restrictive interventions for suicidality must be constantly weighed against the long-term risks. Commonly mentioned risks are increasingly regressive behavior, reinforcement of the patient's hopelessness and avoidance (9), strengthening of the suicidal identity (19), and insufficient treatment of the underlying problems.

Clear, transparent, and warm communication with the patient and the family must be maintained at all times regarding the reasons for not proceeding with a crisis admission or intensification of restrictive safety measures. Ensure that the reasons for decelerating, when not to react to suicidality with restrictive interventions, and when to react are explicitly outlined, documented, and discussed with the patient and the family. If a crisis admission seems inevitable, it is important to determine the purpose, duration (preferably as short as possible), and evaluation timeframes in advance and in conjunction with the patient, the family, and the therapists involved (9).

### 3.2 View suicidality as a coping mechanism

The function and meaning of suicidal behavior should be mapped out in collaboration with the patient and parents. In their differentiation model, de Winter et al. (28) distinguish four subtypes of suicidal behavior with a unique pathway to entrapment, including the type in which the entrapment is originated in the context of communicating about intense suffering. This suicidal behavior is identified as inadequate communication and/or coping. Due to a lack of adequate coping and affect regulation skills, the patient may express severe distress, a cry for help, and the need for a solution through suicidality alone (28). During adolescence, affect regulation is still developing, which may limit the resources of adequate emotional and cognitive coping skills, including seeking social support, expressing feelings towards others, and accurately identifying and accepting emotions. Consequently, adolescents may be more prone to resorting to inadequate coping skills (18).

In the clinical field, it is frequently observed that patients in this subgroup stagnate in their development of identity and appear to be emotionally immature (2). The patient's ability to act autonomously comes under pressure. As the parents of Patient stated in their interview: she became emotionally younger and seemed no longer able to cope with the responsibilities she was expected to bear given her age.

It is important to give attention to the potential discrepancy between the desired social, emotional, and cognitive skills of the patient and their actual capabilities, which can result in sustained and excessive pressure, emotional distress, and burnout. Be alert to the possibility that suicidality may represent an inadequate ability to cope with underlying issues (for example, a way to regulate anxiety) or serve as avoidance behavior from responsibilities and tasks that are perceived as too challenging due to sustained and excessive emotional pressure. We therefore deem it important to create an environment in which the risks of excessive emotional pressure are reduced, so that autonomy can be fostered within a framework of proximity and safety.

### 3.3 Avoid referrals and ensure continuity

Referrals should be avoided and continuity in the treatment process should be ensured by building a multidisciplinary care network around the patient. Due to the often complex comorbidities in chronically suicidal adolescents (5), it is plausible their symptom profiles and healthcare needs fluctuate over time (29). Simply put: as one disorder is treated, another emerges. This is followed by referrals to different healthcare providers, treatment programs, and teams, each with their own specific expertise.

However, a robust network of care should be constructed around these patients, in particular, by bundling expertise and collaborating across care program boundaries in conjunction with the patient, relatives, and the healthcare providers concerned. Responsibility for providing care should be taken collectively. The focus of treatment of a disorder should shift towards treating the overlapping etiological, pathological, and sustaining mechanisms in dysfunction (29).

In our experience, the Dutch F-ACT youth care model aligns well with these recommendations. This care model involves a multidisciplinary team that can offer intensive outreach treatment and practical support for complex and fluctuating care needs across different life domains to both the patient and the family (30).

### 3.4 Aim at support for the treatment considerations at multiple levels

Aim at gaining support for the treatment considerations from the patient's entire network—in other words, the patient themselves, parents, family, (mental) healthcare providers, school, and the general practitioner. Parents and schools play a crucial role in the patient's daily life and may also be part of the mechanisms that explain or sustain the problem. As such, they must be closely involved in the treatment (31, 32). In the case of suicidality, the shift from restrictively oriented treatment to treatment that promotes recovery and autonomy seems to be gaining acceptance among (mental) healthcare providers. However, parents (and society) seem to find it difficult to adopt this way of thinking. The belief that a crisis admission is helpful and effective for treating suicidality is deeply ingrained, despite a lack of evidence to that effect (9, 33).

An understanding of (the function of) suicidal behavior in a broader context and the consequences of restrictively oriented interventions is therefore essential, as is close cooperation and agreement on the treatment considerations with the parties involved. The resilience of parents should be optimized—for example, through psychoeducation, parental guidance, family therapy, and by expanding the support network, as in the case of YST (27).

### 3.5 Make chronic suicidality bearable and tolerable for the mental healthcare provider

Treating chronic suicidality must be made bearable and tolerable for the mental healthcare provider. The experiences of mental healthcare providers often remain unnoticed, while feelings of fear, frustration, incompetence, and despondency in regard to treating patients with suicidality are not uncommon (11, 34).

The advice to decelerate and not react directly to suicidal behavior may go against the natural course of action. The understanding and validating attitude referred to earlier is only possible if the therapist is not overwhelmed by feelings of counter transference. Sharing these feelings in intervision or supervision with the team is essential to prevent acting out feelings of counter transference, and can also help to share the experienced burden (35).

Dialectical behavior therapy, which involves a mandatory weekly consultation team meeting for therapists, ensures both intervision and supervision (36). Moreover, it is not recommended to involve only one therapist in the patient's treatment; rather, the aim should be to achieve shared responsibility with a small team of therapists, in order to guarantee continuity and consistency in the treatment process (30). Moreover, we believe it is of great importance that suicide prevention and the associated treatment dilemmas in the provision of mental health care for chronically suicidal adolescents should be adequately covered in the curriculum for future therapists and psychiatrists.

### 3.6 Use the framework of the Dutch compulsory mental healthcare act

In the Netherlands, the pressure to resort to restrictively oriented interventions (in the form of compulsory care) is often fueled by a sense of duty to act under the Dutch Compulsory Mental Healthcare Act (in Dutch: *Wvvgz*; link for more information: https://www.dwangindezorg.nl/wvggz/english-version). This Act applies to people in whom a psychological disorder results in behavior that can lead to serious harm to themselves or to others. It can only be applied when voluntary care to eliminate serious harm is not possible. However, the Act also offers protection against ineffective treatment and iatrogenic harm. Compulsory care should be a last resort and therefore meet the conditions of proportionality, subsidiarity, effectiveness, and safety. In the subgroup of patients described in this paper, the conditions of effectiveness and safety seem to be compromised. A clinical admission can increase the distance between the patient and their family, potentially reinforcing common cognitions, such as “I am a burden to others”, or “I am insignificant”, as well as feelings of loneliness. Furthermore, compulsory care in this subgroup seems to lead to a confirmation of the core cognition “I can't do it alone”, a growing sense of incapacity, and often a severe regression in suicidal behavior as soon as more responsibility is demanded. If the patient's care is thoroughly discussed, evaluated, and reported in the light of the possible consequences of compulsory care and the conditions of the abovementioned Act, this will stimulate treatment policies that leave autonomy as intact as possible in the case of chronic suicidality. Involving the medical director of the institution in this process can also be helpful.

## 4 Conclusion

In conclusion, the persistent suicidal threat can result in a treatment with considerations primarily focused on managing the suicidality by restrictive safety measures. This may lead to insufficient treatment of underlying issues, ongoing reinforcement of the suicidal behavior, and eventually, a risk of developing a suicidal identity. To address these concerns, we propose treating the chronic suicidality as a transdiagnostic phenomenon, promoting autonomy in treatment, ensuring continuity of care by creating a multidisciplinary network of care providers, and supporting care providers in managing chronic suicidality through supervision and training. It is important to note that these recommendations should be integrated into an ongoing treatment process. They represent merely an initial step towards improving mental healthcare for adolescents with chronic suicidality and require further development and research.

## Statement

This is an English language translation of the Dutch article *Het behandelbeleid van vrouwelijke adolescenten gegijzeld door chronische suïcidaliteit* originally published in *Tijdschrift for Psychiatrie* [In English: *Journal for Psychiatry*; (37)]. Van de Koppel (first author) prepared this translation with support from 113 Suicide Prevention. Permission was granted by Lianne van der Meer, Editor of *Tijdschrift voor Psychiatrie*.

## Data Availability

The data analyzed in this study is subject to the following licenses/restrictions: Interview data cannot be shared publicly because of ethical restrictions: the dataset contains potentially identifying and sensitive information and the Medical Research Ethics Committee (MREC) of Amsterdam UMC has imposed this restriction (registration number: 2018.651—NL68348.029.18). Contact information: https://metc.amsterdamumc.org/contact/. Requests to access these datasets should be directed to Saskia Mérelle, S.Merelle@113.nl.
